# Convergent functional genomics in addiction research - a translational approach to study candidate genes and gene networks

**DOI:** 10.1186/2193-9616-1-18

**Published:** 2013-12-13

**Authors:** Rainer Spanagel

**Affiliations:** Institute of Psychopharmacology, Central Institute of Mental Health, Faculty of Medicine Mannheim, University of Heidelberg, J5, 68159 Mannheim, Germany

**Keywords:** Substance use disorders, Alcohol addiction, Behavioral addictions, Endophenotypes, QTL analysis, Transcriptomic analysis, GWAS, Transgenic animal models

## Abstract

Convergent functional genomics (CFG) is a translational methodology that integrates in a Bayesian fashion multiple lines of evidence from studies in human and animal models to get a better understanding of the genetics of a disease or pathological behavior. Here the integration of data sets that derive from forward genetics in animals and genetic association studies including genome wide association studies (GWAS) in humans is described for addictive behavior. The aim of forward genetics in animals and association studies in humans is to identify mutations (e.g. SNPs) that produce a certain phenotype; i.e. “*from phenotype to genotype*”. Most powerful in terms of forward genetics is combined quantitative trait loci (QTL) analysis and gene expression profiling in recombinant inbreed rodent lines or genetically selected animals for a specific phenotype, e.g. high vs. low drug consumption. By Bayesian scoring genomic information from forward genetics in animals is then combined with human GWAS data on a similar addiction-relevant phenotype. This integrative approach generates a robust candidate gene list that has to be functionally validated by means of reverse genetics in animals; i.e. “*from genotype to phenotype*”. It is proposed that studying addiction relevant phenotypes and endophenotypes by this CFG approach will allow a better determination of the genetics of addictive behavior.

## Review

According to the World Health Organization (WHO), approx. 2 billion people drink alcohol, 1.3 billion people use tobacco, and almost 200 million people use illicit drugs. Many of those alcohol, tobacco and drug users continue taking these substances despite developing severe health and social problems and a substantial proportion become addicted. Drug addiction^a^ is defined as a pathological behavioral syndrome with compulsive drug use, craving, and relapses that can occur even after years of abstinence. There are different classes of substances including alcohol, nicotine, cannabis, opiates, and stimulants. All of them can lead to addictive behavior.

Very recently, the term addiction has been applied to a range of problematic behaviors such as pathological gambling and pathological internet use, to mention only a few. Consequently, the new psychiatric classification system DSM-5 for the first time attempts to categorize substance use disorders *vs.* so-called behavioral addictions. The DSM-V committee decided to accept “Gambling Disorder” (or pathological gambling) as an addiction and to put “Internet Use Disorder” into the category, where more research is needed.

One fundamental question in addiction research is: What are the genetic factors underlying this pathological behavior and to which extent do alcohol, nicotine, opiate, cannabis, and cocaine addiction and also behavioral addictions share genetic mechanisms? Knowledge about distinct and shared genetic mechanisms of substance use disorders (SUDs) has important implications for diagnosis, treatment, addiction theories and future research.

### Genetics of SUDs, alcohol addiction and behavioral addictions

Twin, adoption and sibling studies have shown that genetic influences are directly responsible for some of the inter-individual differences observed in the predisposition to addictive behavior. A meta-analysis that included several sets of ten thousands of monozygotic and dizygotic twin pairs, estimated a heritability of different drug addictions to lie at around 40-70% (Goldman 2005). There have also been two major twin studies of pathological gambling with consistent evidence for heritable variation (50%) (Slutske et al. 2010; Agrawal et al. 2012). Typically any form of addiction is a complex disorder that shows no obvious Mendelian transmission pattern and provides no evidence for main gene effects. Thus the contribution of single genes to the clinical phenotype, perhaps with the exception of some rare variants (Malhotra and Sebat 2012), is rather small.

Does a genetic overlap exist between different drugs of abuse? Family studies have revealed that across several drug classes (opioids, cocaine, cannabis, nicotine, alcohol), the offspring of substance abusers are at 2 to 8-fold increased risk to develop an addictive behavior (Merikangas et al. 1998; Merikangas and McClair 2012). A third of the variance in risk for nicotine and cannabis addiction, and about 40% of the variance in alcohol addiction is accounted for by additive genetic factors common to all three disorders (Xian et al. 2008; Palmer et al. 2012). Furthermore, it has been shown that there also exits a genetic overlap between drug and behavioral addictions; e.g. 20% of the genetic risk for pathological gambling has been shown to be accounted for by the genetic risk for alcohol addiction (Slutske et al. 2000; Lobo and Kennedy 2009).

Technological advancements such as next generation sequencing and systematic genome wide association studies (GWASs) play a crucial role in candidate gene discovery today. These technological developments also led to a sharp increase in publications on the genetics of addiction in the last decade (Helinski and Spanagel 2011) and will help to identify shared and distinct gene patterns associated with different drug addictions and behavioral addictions. Rare functional exonic variants can now be efficiently genotyped, allowing exome-wide association tests but detection of individual variants may require very large samples (Vrieze et al. 2013). Nevertheless, in a quiet small sample of cases and controls, deep resequencing of glutamate system genes allowed in a very recent study the identification of several rare variants affecting risk of opioid dependence demonstrating that depending on the hypothesis exome sequencing can yield significant results even in less than 1000 affected cases (Xie et al. 2013).

GWASs play a crucial role in candidate gene discovery today and have been successfully applied to addiction research, especially in nicotine and alcohol addiction where meta-analyses with over 80,000 individuals of European ancestry are available today.

GWAS of smoking behavior and nicotine addiction have produced consistent and compelling genetic evidence for association (Bierut et al. 2008). The strongest genetic contribution to nicotine addiction comes from variation in the nicotinic acetylcholine receptor subunits. The most robust genetic finding that alters the risk of developing heavy smoking and nicotine addiction is in the chromosome 15q25 region, which contains the α5, α3, and β4 nicotinic receptor subunit gene cluster (*CHRNA5, CHRNA3, CHRNB4*). The SNP rs16969968 is unequivocally associated with smoking behavior (p=5.6 × 10^−72^) (Tobacco and Genetics Consortium 2010).

**Figure 1:**
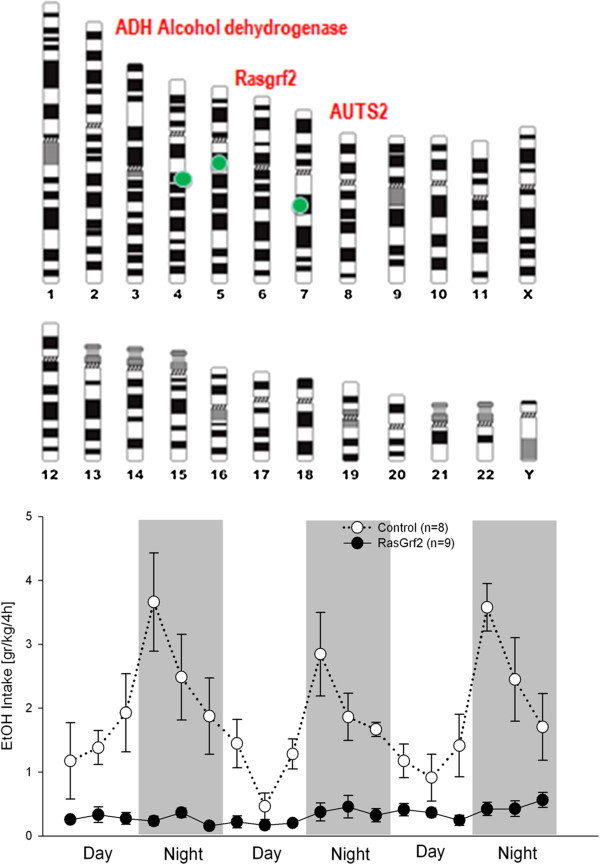
**Genome wide significant findings for alcohol consumption and alcohol addiction.** Alcohol metabolizing genes, especially in the *ADH* cluster, are consistently found to be associated with alcohol addiction. Meta-analysis on large population based samples with almost 50,000 individuals demonstrated that a single nucleotide polymorphism (SNP) (rs6943555) in *autism susceptibility candidate 2* gene (AUTS2) was associated with alcohol consumption at genome-wide significance (Schumann et al. 2011). This finding was supported by multiple lines of evidence from mouse and drosophila studies. Convergent evidence was also obtained for association of SNP rs26907 in the *ras-specific guanine-nucleotide releasing factor 2* (RASGRF2) gene with alcohol consumption (Stacey et al. 2012). This finding was functionally validated in Rasgrf2 knockout mice – this is shown in the lower panel where alcohol consumption in knockout and control littermate mice was measured in a drinkometer system (Vengeliene et al. 2013) throughout day and night time. Rasgrf2 knockouts show completely blunted alcohol consumption confirming the human findings that RASGRF2 is critically involved in regulating alcohol consumption (drinking data were kindly provided by Ainhoa Bilbao).

Fifteen GWASs of alcohol dependence and symptoms of alcohol dependence, and nine studies of alcohol consumption and other alcohol dependence related traits have to date been published (Rietschel and Treutlein 2012). The first GWAS on alcohol addiction (Treutlein et al. 2009) and alcohol consumption (Schumann et al. 2011; Stacey et al. 2012) yielded several genome-wide findings, especially in the alcohol dehydrogenease (*ADH*) cluster (Frank et al. 2012) and *AUTS2* (Schumann et al. 2011) and were replicated in independent studies (Biernacka et al. 2013 (*ADH*, Kapoor et al. 2013 (*AUTS2*)) (Figure 1).

 (Agrawal *et al.*^2011^) reported the first genome-wide association study (GWAS) of cannabis addiction in 708 cannabis dependent and more than 2000 control subjects. None of the association signals reached genome-wide significance most likely due to the small sample size. In a larger study (Verweij *et al*. 2012) several previously identified candidate genes for cannabis use were studied in 7452 families from the Australian Twin Registry but none of the 10 candidate genes were associated with lifetime cannabis use. Very recently a meta-analysis with more than 10,000 individuals was performed with again no genome-wide findings. Even gene-based association test in this large sample of cannabis abusers revealed no significant effects of individual genes (Verweij *et al*. 2013). However, it should be mentioned that the gene (CNR1) that encodes the cannabinoid receptor 1 plays a moderate role in modulating addictive behavior across several SUDs (Benyamina et al. 2011).

Gelernter et al. (2013a) reported on the first GWAS in opioid addicted patients with genome wide significant findings with genes involved in potassium signaling pathways. Pathway analysis also implicated genes involved in calcium signaling and long-term potentiation. The same authors (Gelernter et al. 2013b) also reported on the first GWAS in cocaine addiction in three sets of African- and European-American subjects and identified several risk variants. A first GWAS of pathological gambling was also recently conducted (Lind et al. 2013). While further replication is required, the identification of susceptibility loci and biological pathways will be important in characterizing the biological mechanisms that underpin pathological gambling.

All GWAS-derived findings in addiction research are summarized and constantly updated in the Addiction GWAS Resource (AGR; addictiongwas.com; Spanagel et al. (2013)). Although GWAS data sets are available now for all different drug addictions and also for pathological gambling, the data sets most likely do not contain enough power to estimate shared and distinct genetic components for these disorders. Hence many more GWASs have to be performed to make comparative analyses across different SUDs, alcohol addiction and behavioral addictions.

Besides GWAS, genetic system-level approaches were successfully used to estimate the overall effect of genetic variations on a specific phenotype, for example within a specific neurotransmitter system on alcohol drinking. In particular, genetic system-level approaches in the monaminergic (Chen et al. 2012; Clarke et al. 2012; Filbey et al. 2012), glutamatergic (Schumann et al. 2008; Karpyak et al. 2012) and opioidergic (Levran et al. 2012; Bazov et al. 2013) systems support a role of these neurotransmitter/peptide systems in both alcohol drinking and addictive behavior. Those prior knowledge driven genetic system-level approaches do not necessarily overlap with gene network analyses which are used to find modules of highly co-expressed genes with a gene of interest. The generally held view is that genes which are associated or interacting are more likely to share function and thereby build up a network. However, this view seems to be the exception rather than the rule in gene networks (Gillis and Pavlidis 2012) since functional information within gene networks is typically concentrated in only a very few interactions whose properties cannot be reliably related to the rest of the network. Thus gene function is not necessarily encoded in a whole gene cluster.

Finally, SUDs and alcohol addiction are the result of cumulative responses to drug/alcohol exposure, the genetic make-up of an individual, and environmental perturbations over time (Spanagel, 2009). Understanding how environmental influences moderate genetic risk (gene x environment interactions; G x E) is crucial for the elucidation of mechanisms underlying these disorders (Sher et al. 2010; Spanagel et al. 2010; Yan et al. 2013). In the context of G x E interactions, epigenetic mechanisms may also substantially influence the initiation of addictive behavior, and the first whole genome DNA methylation map has very recently been obtained from alcohol-dependent patients (Zhang et al. 2013) along with the demonstration of elevated methylation in the brain of alcoholics (Taqi et al. 2011). Furthermore hypermethylation of genes (e.g. the DAT gene locus) in alcohol-dependent patients seems to be the consequence, rather than a cause, of the disorder (Nieratschker et al. 2012).

In summary, although some genetic variants in candidate genes have been convincingly identified by GWASs the functional role of these gene variants in the development and maintenance of addictive behavior, with some exceptions such as the alcohol metabolising *ADH* variants, are poorly understood. Furthermore, only a small percentage of the variation in drug / alcohol use initiation and addictive behavior is due to common genetic variants and most likely hundreds and more probably thousands of genetic variants will be required to fully explain the genetic input to drug addictions and behavioral addictions (Bierut 2011). But even if we will identify by larger and larger samples more and more risk variants we still do not understand the interactions of these risk variants with environmental factors such as stress and their epigenetic modulation (Tsankova et al. 2007).

Here I propose to use a convergent functional genomics (CFG) approach (Niculescu and Le-Niculescu 2010) with the entire spectrum of state-of-the-art methodologies to study the genetics of addictive behavior. Although a CFG approach will not yield more candidate genes than a classical GWAS it will provide convergent evidence from several lines of genetic analysis – beyond the “p-value hysteria and illusion” of human genetics today – to functionally pin down in a convincing manner risk genes and gene networks involved in addictive behavior. In the following I will briefly outline in a general way the CFG approach, and will then describe the “ingredients” for future, hopefully successful CFG studies in the addiction field.

### The convergent functional genomics (CFG) approach

The term “convergent functional genomics” (CFG) has been coined by Alexander B. Niculescu (Niculescu et al. 2000). It is an approach for identifying and prioritizing candidate genes for complex psychiatric disorders (and other diseases) by integrating multiple lines of evidence – e.g. gene expression and genetic data from human studies and animal model work. The more lines of evidence for a gene, the higher it comes up on the CFG prioritization list. This is similar conceptually to the Google PageRank algorithm, in which the more links to a page the higher it comes up on the search prioritization list. In a CFG approach biologically-relevant signal even from limited size studies are extracted and prioritized in a Bayesian fashion. According to Bayesian theory, an optimal estimate results from combining previous information with new evidence. Although one cannot exclude that some of the candidate genes that will be identified through this approach are false positives because of potential biological or technical limitations of the methodology and approach employed, logically the higher the number of independent lines of evidence (i.e. the higher the CFG score), the lower the likelihood of that being the case. Thus at the end of the day such an approach results in a polyevidence CFG score. It is obvious that the way of weighing the lines of evidence is on a subjective base (usually between 0.5-1 score per line of evidence) and may thereby give slightly different results in terms of prioritization, if not in terms of the list of genes per se (Niculescu and Le-Niculescu 2010). Nevertheless, the calculation of CFG scores for example for schizophrenia (Ayalew et al. 2012), bipolar disorder (Patel et al. 2010) and anxiety disorders (Le-Niculescu et al. 2011) resulted in plausible and reproducible ranked candidate gene lists.

What is important however is the functional validation of candidate genes. Here I propose to functionally validate candidate genes by reverse genetics; i.e. to use a conventional rodent knockout model that lacks the gene of interest. More advanced transgenic models that lack for example the gene of interest in a specific neuronal population (Bilbao 2013) are less suited in this respect because a risk allele derived either from a GWAS or a CFG approach is usually expressed in all cells. Alternatively humanized rodent models can be used for validation. Here the risk as well as the non-risk allele can be expressed in the mouse or rat genome and then studied for their drug-related phenotypes (Ramchandani et al. 2011) and the development of addictive behavior. Compared to other psychiatric research fields, drug addiction researchers are in a good position to test those transgenic animals as DSM-based animal models with excellent face, construct and predictive validity are available (Sanchis-Segura and Spanagel 2006; Cannella et al. 2013; Deroche-Gamonet and Piazza 2013; Vengeliene et al. 2014).

### CFG application to addiction research – first step: providing accumulated evidence for candidate genes from forward genetics

Niculescu and collaborators also applied a CFG approach to alcoholism (Rodd et al. 2007). In this study they converged multiple independent internal (gene expression profiling in genetically selected rats for high vs. low alcohol preference) and external lines (mainly external databank and literature-based information) of evidence for Bayesian crossvalidation. Highest CFG scores where obtained for alcohol metabolizing genes which is in line with GWAS derived findings (see previous paragraph). Some of the pathways identified in this study even suggest avenues for pharmacotherapy of alcoholism with existing agents, such as angiotensin-converting enzyme (ACE) inhibitors. Indeed the authors could also show that an ACE inhibitor results in strong modulation of alcohol intake in alcohol-preferring rats (Rodd et al. 2007).

Convergent approaches were also used in other studies. Although these approaches generated convergent evidence from different human and animal data sets they did not calculate a CFG score. One good example is the first GWAS on alcohol addiction where the human data set was combined with gene expression data that derived from a DSM-based animal model which allows separating alcohol addicted from non-addicted rats (Vengeliene et al. 2014). This combined data set was subjected to a replication study and eventually led to an enriched candidate gene list (Treutlein et al. 2009).

As said, here I want to propose a CFG approach based on the work of Alexander Niculesu and collaborators that uses the entire spectrum of state-of-the-art methodologies of forward genetics. The repertoire of forward genetics includes many different approaches and I will only refer to some of them. Most straightforward in terms of forward genetics is QTL^b^ analysis in inbreed rodent strains, especially in recombinant strains (lines) combined with gene expression profiling (Spence et al. 2005; Crabbe et al. 2010). This can be also done in genetically selected animals for a specific phenotype, e.g. high vs. low drug consumption (Spence et al. 2009; Crabbe et al. 2010).

A recombinant inbred strain (line) is an organism with chromosomes that incorporate an essentially permanent set of recombination events between chromosomes inherited from two or more inbred strains. F1 and F2 generations are produced by intercrossing the inbred strains; pairs of the F2 progeny are then mated to establish inbred strains through long-term inbreeding. Families of recombinant inbred are then used to map the locations of DNA sequence differences (QTLs) that contribute to differences in a particular phenotype of interest; e.g. alcohol consumption. The larger the family of recombinant inbred strains, the greater the power and resolution with which phenotypes can be mapped to chromosomal locations. Of particular interest for addiction researchers is the BXD family of recombinant inbred strains which was derived by crossing C57BL/6J and DBA/2J and inbreeding progeny for 20 or more generations (http://www.genenetwork.org). With this resource data for hundreds of phenotypes have been acquired over a nearly 40-year period. In particular, the BXD panel is used to study the genetics of behavioral phenotypes of alcohol and drug addiction, stress, and locomotor activity (Hoffman et al. 2011; Wang et al. 2012). Another advantage of the BXD family of strains is that the both parents have been sequenced and these two strains differ at approximately 4.8 million SNPs (http://www.genenetwork.org). Thus variants (mostly single nucleotide polymorphisms and about 500,000 insertion-deletions) that produce interesting phenotypes can be located efficiently. Importantly, large gene expression data sets for different brain regions of the BXD strains are also available (Wang et al. 2012). This large database on BXD strains allows not only mapping new drug-related phenotypes to specific expression QTLs but also to examine GWAS derived candidate genes.

Alternatively, genetically selected animals can be used for combined QTL and expression analyses. In particular, inbreed alcohol-preferring (iP) and -nonpreferring (iNP) lines were developed from Wistar rats to model high and low voluntary alcohol consumption, respectively and to perform genetic studies (Crabbe et al. 2010). Using iP and iNP strains, a strong QTL for alcohol consumption was identified on rat chromosome 4 (Spence et al. 2009). To search for candidate genes that underlie this chromosomal region, complementary molecular-based strategies were implemented to identify genetic targets that likely contribute to the linkage signal. Thus far, three candidate genes, neuropeptide Y (NPY), alpha-synuclein, and corticotrophin-releasing factor receptor 2, have been identified that may account for the linkage signal (Spence et al. 2009). The essential role of NPY in regulating alcohol consumption and other alcohol-related behaviors has been convincingly be demonstrated by the use of knockout and NPY overexpressing mice (Thiele et al. 1998; Hayes et al. 2012).

Very recently it was found that alcohol-preferring P rats are homozygous for a Grm2^c^ stop codon that leads to largely uncompensated loss of mGluR2 (Zhou et al. 2013). In this study the stop codon variation was linked to increased alcohol consumption and preference in F2 rats generated by intercrossing iP and iNP rats. The causal role of mGluR2 in altered alcohol preference was finally confirmed by elevated alcohol consumption in Grm2 knockout mice. Interestingly, in the brain of alcoholic patients a strong down-regulation of Grm2 transcripts has also been found (Meinhardt et al. 2013). Together, these data point to mGluR2 as an origin of alcohol preference and a potential therapeutic target and demonstrate how powerful genetic analysis in inbred alcohol preferring animals can be.

Gene expression profiling studies in brain areas which are of relevance for mediating alcohol and drug-induced effects such as reinforcement (Noori et al. 2012) in DSM-based animal models can also yield candidate gene lists that can be used for CFG scoring. For example gene expression profiling in the dorsal striatum of alcohol addicted vs. non-addicted rats showed an up-regulation of dopamine D3 receptor transcripts and in a series of follow-up studies the critical role of D3 receptors in addictive behavior was confirmed (Vengeliene et al. 2006; Song et al. 2012; Xi et al. 2013).

Beside the here described forward genetic approaches other omics based approaches and in particular proteomics (Wang et al. 2011) can be used to achieve convergent evidence for the role of a particular gene in a drug-related phenotype of interest. These lines of evidence derived from animal work can be used to calculate a CFG score without giving any particular weight to one or another approach; i.e. each line of evidence for a particular gene of interest would be scored by one. This animal research based CFG score would then be added to the lines of evidence obtained on the human side. Here as already mentioned linkage data, GWAS data, gene expression data from brain and other tissues, and also proteomics data can provide in addition to the animal data polyevidence for a gene/gene product of interest. In conclusion, candidate genes derived from a CFG approach can be categorized according to their CFG score into top candidate genes and categories of less importance.

### Second step: functional validation of candidate genes by reverse genetics

It is increasingly evident that functional validation of the role of genes identified by forward genetic models is a critical step of strengthening the case for a causal relationship between the gene and the observed phenotype. One way of validation is to manipulate the gene using reverse genetics and test the manipulated animals in behavioral tests or models (Sanchis-Segura and Spanagel 2006). The choice of strategy for doing this may depend on a lot of factors such as the level of existing knowledge about the gene. One important issue is the choice of species. In a bigger perspective this is often a tradeoff between similarity to humans and practical issues regarding maintaining the species in an animal facility (size, length of reproductive cycle, aggressiveness and cost). A particular important question in the context of reverse genetics is also the availability of genetic models in the chosen species. Today, the mouse is the standard species for studying drug addiction with reverse genetics. However, rats are becoming increasingly popular and this trend will most likely continue since it seems like the rat is a better choice for complicated long-term models of addiction (Kasanetz et al. 2013; Cannella et al. 2013; Vengeliene et al. 2014). Further, several genetic techniques now can be used in the rat (Geurts et al. 2009; Tong et al. 2010; Brown et al. 2013). However, the mouse still has the advantages of the plethora of lines available, a more widespread availability of facilities and laboratories with the capacity to generate mutant lines and the lower cost per animal (which is a critical factor in models requiring a lot of breeding).

Reverse genetics can be broadly divided into approaches resulting in random integration of transgenes and targeted approaches. The first category includes “classic” transgenes introduced by pronuclear injection and a variety of viral-mediated techniques. These approaches have the advantage of being relatively rapid. The use of constructs based on bacterial artificial chromosomes has become increasingly popular during the last decade since it makes the expression of transgenes made by pronuclear injection much more reliable (Heintz 2001). The targeted approaches include the generation of knockout and knockin animals. The latter is particularly interesting as a functional validation of data from humans (e.g. GWAS data) since it can be used to modify an allele in the mouse to mimic an allele known to affect the risk of a specific phenotype in man.

Expression of dominant negative molecules is another alternative for inhibiting the function of certain molecules (Heusner and Palmiter 2005). This approach can also be used to silence families of molecules (e.g. an ion channels subunit engineered to block the pore will block all channels where it is integrated independent of subunit composition). Another way of silencing genes is the use of gene-targeted zinc finger nucleases. This methodology has been used to inactivate genes in a sequence specific manner in the rat (Geurts et al. 2009). Recently, it has also been used to mediate homologous recombination in mouse zygotes, showing that it has potential beyond simple inactivation (Meyer et al. 2010). Another interesting resource is repositories with ES cell lines in which genes have been inactivated by gene trapping (Guan et al. 2010). Gene trapping has also been used to generate conditional alleles, expanding the use of these repositories. In addition to gene trap based repositories there are other initiatives for providing researchers with classic knockout mice and mice with conditional mutations on a genome-wide level (Guan et al. 2010). Together with the accumulating number of mutant mice generated by research groups all over the world it will be much easier to find already existing mutant mice for studying the candidate gene of interest.

## Conclusion

GWASs in the addiction field provided first interesting insights into the genes that drive, at least in part, a drug-related phenotype. However, given that much of the variance is driven by societal, lifestyle and behavioral influences - and in addition there are also problems related to DSM-based diagnostic criteria (Miller 2010) - larger sample sizes for GWA analysis, inclusion of endophenotypes and CFG approaches are warranted. Having already massive transcriptomic, genetic and phenotypic datasets available a Bayesian-like integration strategy can be applied where multiple independent lines of genetic and genomic evidence is used, each by itself lacking sufficient discriminatory power, but combined leads to the identification of high probability candidate genes or gene clusters. The role in the etiology of addiction of these high probability candidate genes or gene clusters and their interaction with environmental factors will have to be finally validated in appropriate knockout and humanized animal models.

## Endnotes

^a^The term “drug addictions” used in this review is the same as the DSM-5 term “substance use disorders”.

^b^A mapped QTL defines the location of a gene (or more than one gene) that influences a complex trait (i.e., one that is influenced by multiple genes, by environmental factors and can be influenced by all possible interactions of these variables).

^c^Grm2 encodes metabotropic glutamate receptor 2 (mGluR2) which is critically involved in regulating extrasynaptic glutamate levels.
